# Suicide Risk Following Psychiatric Hospitalization: A Narrative Review and Conceptual Synthesis

**DOI:** 10.3390/ijerph23050587

**Published:** 2026-04-30

**Authors:** Evelien van Gelderen, Rebecca Marquard, Olivia E. Nasef, Robert L. Bogue, Paul S. Nestadt

**Affiliations:** 1 Johns Hopkins School of Medicine, Baltimore, MD 21287, USA; evangel1@jhmi.edu (E.v.G.); onasef1@jh.edu (O.E.N.); 2 Department of Psychiatry, University of Maryland, Baltimore, MD 21201, USA; rebecca.marquard@som.umaryland.edu; 3 Robust Futures, Inc., Carmel, IN 46032, USA; rob.bogue@thorprojects.com; 4 Center for Suicide Prevention, Johns Hopkins Bloomberg School of Public Health, Baltimore, MD 21287, USA; 5 Department of Psychiatry and Behavioral Sciences, Johns Hopkins School of Medicine, Baltimore, MD 21287, USA

**Keywords:** post-discharge suicide, post-hospitalization suicide, suicide intervention, suicide risk, crisis interventions

## Abstract

**Highlights:**

**Public health relevance—How does this work relate to a public health issue?**

- Suicide is a public health crisis with US rates steadily rising, costing around 50,000 lives and approximately $500 billion annually.
- Patients are at the highest risk for suicide immediately following discharge from the hospital.

**Public health significance—Why is this work of significance to public health?**

- There is little supporting evidence for an accurate method to stratify risk.
- The risks and benefits of hospitalization, our highest level of intervention, require careful consideration.

**Public health implications—What are the key implications or messages for practitioners, policy makers and/or researchers in public health?**

- We must consistently opt for the least restrictive care setting possible, while maintaining safety.
- Proposed interventions to reduce suicide risk are varied and need further study.

**Abstract:**

Suicide is a global and public health crisis that impacts people of all ages and backgrounds. The literature supports that individuals with serious mental illness are at a higher risk of suicide compared with those without a serious mental illness. It is also well-documented that individuals are at particularly high risk of suicide immediately post-hospitalization for a psychiatric illness. Our narrative review synthesizes and analyzes the existing literature on this phenomenon, the rates of suicide post-hospitalization, the risk factors for suicide during that time, and the interventions and strategies developed to reduce the rates. Current risk assessments struggle to identify individual patients who are at the highest risk of suicide post-discharge. Research has shifted towards focusing on brief crisis interventions to target this high-risk period. Other interventions in the literature include those that attempt to increase engagement with mental health services and increase institutional resources. We also synthesize literature on the iatrogenic risk of hospitalization, the impact hospitalization itself can have on patients, and their risk of suicide once discharged. Future directions could include further exploration of the impact these interventions have on specific populations, such as those with comorbid psychiatric and substance use conditions.

## 1. Introduction

Suicide is a public health crisis and global phenomenon. According to the World Health Organization, it is the third leading cause of death among 15–29 year olds and impacts people all around the world. In 2021, 73% of global suicides occurred in low and middle-income countries [1]. Global suicide rates have slightly decreased in the past 20 years. In contrast, the US suicide rate has increased since 2000, jumping from 10.7 deaths per 100,000 standard population in 2001 to 14.1 deaths per 100,000 standard population in 2021 [2]. The increasing prominence of psychiatric illness in the US is a likely cause of the increase in suicide rates. It is well-documented in the literature that individuals with serious mental illness are at a higher risk of suicide compared to individuals without a serious mental illness [3,4,5]. A phenomenon that is also well-established is that the first few months after discharge from a psychiatric hospital is a time of particular vulnerability to suicide and suicide attempts [6,7]. Thus, we found it crucial to summarize, analyze and integrate studies that investigated the rates of suicide post-hospitalization, risk factors for suicide in that time, and potential interventions and strategies. Given the extent of the impact of suicide on worldwide mortality, understanding and addressing this period of high-risk may allow clinicians to tailor their interventions and ultimately save lives.

## 2. Methods

We conducted a narrative review and conceptual synthesis of the literature examining suicide risk following discharge from psychiatric hospitalization. The purpose of this review was not to undertake a systematic or exhaustive assessment of the literature, but to integrate and interpret findings across epidemiologic studies, reviews, and selected primary investigations. We aimed to characterize patterns of post-discharge risk, limitations of existing risk stratification approaches, and emerging strategies for prevention during this high-risk period.

A targeted literature search was conducted using PubMed and related databases to identify relevant systematic reviews, meta-analyses, and narrative reviews addressing post-discharge suicide risk, epidemiology, and preventive interventions. These secondary sources were used to anchor the synthesis and to identify frequently cited primary studies that have informed current understanding of the post-discharge period. In addition, selected primary studies were included when they were methodologically influential in foundational study design, widely referenced in the literature with a sustained impact on the field, or illustrative of distinct categories of post-discharge interventions or conceptual approaches.

Given the substantial heterogeneity in study populations, outcome definitions, follow-up intervals, and analytic methods across this literature, we did not apply formal inclusion or exclusion criteria or conduct a quantitative assessment of study quality. Additionally, because this is a systematic review, we intentionally chose not to apply formal inclusion or exclusion criteria. Instead, studies were considered based on their relevance to post-discharge suicide risk and their contribution to understanding epidemiologic patterns, limitations of prediction, iatrogenic factors associated with psychiatric hospitalization, or intervention strategies. Thus, we prioritized studies that allowed us to comprehensively explore the different aspects of post-hospitalization suicide risk and strategies to mitigate it, regardless of the country of origin, date published, or publication type.

Findings from the identified literature were synthesized thematically, with attention to the timing and magnitude of post-discharge suicide risk, the performance of commonly used risk assessment approaches, system-level contributors to risk, and interventions designed to mitigate risk during this vulnerable period. Individual studies discussed in the Results are presented as illustrative examples rather than as a comprehensive or comparative evaluation of all available evidence. This narrative approach was selected to facilitate integration of diverse forms of evidence and to support clinically and public health-relevant interpretation rather than formal quantitative pooling.

## 3. Results

In the sections that follow, we synthesize findings across the literature into several recurring themes related to post-discharge suicide risk, its determinants, and evaluated interventions.

### 3.1. Evidence of the Problem

Multiple studies have found that patients with a psychiatric disorder are at higher risk for suicide after discharge from psychiatric facilities than patients without a psychiatric disorder, and that the rate of suicide post-discharge is mediated by diagnosis and decreases over time. As an illustrative example, a meta-analysis estimated post-discharge suicide rates in the first week are at 3000 per 100,000 person-years and in the first month, 2000, roughly 200–300 times the global average [8]. These figures reflect annualized rates expressed as patient-years despite being derived from short post-discharge intervals. Another cohort study found that the suicide risk for patients 1 year after discharge from a psychiatric hospital was significantly higher compared with the general population (overall SMR for premature death was 7.5, 95% CI 7.2–7.9) [9]. This finding could likely be interpreted as the psychiatrically hospitalized population carries diagnosed psychiatric illness and greater clinical severity than the general population, rather than hospitalization itself being the sole driver of elevated suicide risk. This has also been found in studies that investigate populations in other countries; a study conducted in Korea found that compared to the general Korean population, the suicide mortality rate was 82-fold higher for suicide attempt patients who were admitted to the hospital (SMR 82.0, 95% CI 35.3–161.7) [10]. In a population-based study in Oxford, UK, the suicide rate in the first 28 days after discharge was 213 times more common for male patients and 134 times more common for female patients than would be expected in the general population [11].

Extensive data show that the suicide risk for patients with a psychiatric disorder is particularly high in the period after discharge and declines as time progresses. A meta-analysis found that the post-discharge suicide rate was 100 times the global suicide rate in the 3 months after discharge [6]. One retrospective case–control study of 100 psychiatric English patients found that 55% of suicides took place within a week of discharge, and 49% of the cohort had died before their first follow-up appointment, highlighting how the time immediately after discharge is a particularly vulnerable one [12]. Another study found that suicide risk was higher in patients who received less than the median duration of hospital treatment and that the suicide risk peaked in the first week after admission and in the first week after discharge [13]. In a study of discharges from all psychiatric hospitals or psychiatric wards in Hong Kong from 1997 to 1999, the authors found that 30% of suicides occurred within 28 days after discharge [14]. Looking longer term, one study conducted in Republic of Korea found that the suicide rate declined over time after discharge, and was highest in the first year after discharge at 36.7% of the total suicides of the cohort within 7 years [15].

Multiple studies have investigated which specific populations are most at risk for suicide after discharge. One meta-analysis found that the suicide rate was lower among adolescents than in samples of adults and found no difference in suicide rates between females and males [6]. Other studies have investigated which particular psychiatric disorders place patients at higher risk for suicide after discharge. For example, one study of 1.9 million adult inpatients in the Medicaid program found that the suicide risk during the first 90 days after being discharged was highest for patients with depressive disorders, bipolar disorders, schizophrenia, and substance-use disorders, and was much higher than corresponding rates for the cohort with non-mental disorders [16]. Another study that looked at individuals discharged from psychiatric hospitals in Sweden found that the risk of suicide was increased for patients with schizophrenia if they had engaged in a recent self-harm event before admission, and patients with depression had the highest overall risk of suicide post-discharge, defined as 30 days after discharge from the hospital [17]. These studies suggest that patients with depressive disorders and schizophrenia who engage in self-harm may be at the highest risk for suicide in the weeks after discharge.

Accurately stratifying risk has been proposed as an important step in preventing suicide deaths, although there is a lack of data supporting that there is an accurate way to do so. Standardized suicide risk assessment tools have become common practice in many psychiatric and medical settings, with national governing bodies such as the Joint Commission and Surgeon General calling for more regular and standardized suicide risk assessments [18]. In Riblet et al.’s review of root cause analysis for suicide within 7 days of discharge from an inpatient psychiatric facility, a significant contributor to suicide deaths was a lack of standardized risk assessment before discharge [19]. However, even when completing a risk assessment, results can be inaccurate and misattribute risk. A 2011 meta-analysis by Large et al. emphasized this problem of misattribution. When developing a predictive model of suicidality, they found that 97% of patients categorized at high risk of suicide would not go on to die by suicide, while 60% of patients who did complete suicide would have been categorized as low risk [20]. Additional retrospective studies have verified this finding. Choi et al. found that individuals who presented to the emergency department for a suicide attempt and were subsequently discharged from the ED had a 54-fold higher standardized mortality ratio than the general public, highlighting the need for more reliable forms of risk stratification (SMR 54.3, 95% CI 6.1–195.9) [10]. There is ongoing research on the prediction of future suicidal behavior post-hospitalization; one example is the Suicide Crisis Inventory [21]. A more accurate understanding of risk would allow for targeting interventions and alleviating some of the high morbidity and mortality that suicidal behavior contributes to globally. Of note, there has also been an investigation into “warning signs,” which may indicate a “suicide crisis” and imminent risk for suicide, as opposed to risk factors, which could enhance overall detection and intervention of suicidal behavior [22]. Other forms of risk evaluation have also been proposed, such as the “Normic model of risk,” which predicts a death by suicide by seeing how this act would align with the normalcy of their behavior; however, most institutions continue to assess risk based on defined modifiable and non-modifiable risk factors [23].

### 3.2. Identified Risk Factors

The literature has helped define demographic groups who have consistently been shown to have increased risk, considered “static” risk factors in commonly used risk assessment tools. This group is defined by characteristics that cannot be intervened on, such as gender [16,24], race [25], age [16], family history of suicide attempts, personal history of prior suicide attempts or self-harm [19,20], adverse childhood events [25], and marital status. However, this data is often based on large populations of patients receiving care in community settings, and not specifically on inpatients or recently discharged individuals. Large et al. looked specifically at the post-hospitalized population and found that they were not significantly more likely to be single, unemployed, to have serious physical illness, schizophrenia, bipolar disorder, or substance abuse; many characteristics that would have deemed them “high risk” according to traditional models [20]. This raises concerns that the commonly assumed static risk factors may not be applicable when used for patients who have recently been discharged from an inpatient setting. They found that no single factor was strongly associated with suicide in the year after discharge.

As static risk factors cannot be intervened on by clinicians, much focus has been on defining modifiable risk factors, otherwise known as “dynamic.” This includes a variety of characteristics such as diagnosis, social support, and access to psychiatric care. The same study by Large et al. identified two specific dynamic risk factors: unplanned discharge (OR = 2.44) and recent social difficulty (OR = 2.23). Similar findings were noted by Riblet et al., where in a retrospective analysis of those who died by suicide, 50% of the cohort had died by suicide before their follow-up visit, which was scheduled to be within 7 days of discharge, and 20% had either not shown up or canceled their appointment [19]. Within the same study, unplanned discharges (including ‘against medical advice’ discharges and patient-initiated discharges) were the single biggest root cause identified in the study, attributed in part to follow-up care not being arranged. Interestingly, Large et al. found that patients who had less contact with services after discharge were significantly less likely to commit suicide; however, this was attributed to the correct identification of people with a lower risk of completing suicide and not a causal relationship between increased care and increased risk. It appears that the literature supports the need for increased care in the community during such a high-risk time, and as such, there have been many interventions developed to directly target these risk factors.

### 3.3. Iatrogenic Risk of Psychiatric Hospitalization

Another area of discussion in the literature is whether psychiatric hospitalization itself can increase the risk of suicide attempts among patients [26]. Some scholars note that inpatient treatment itself can be damaging, including being perceived as stigmatizing, coercive, and traumatic, and can isolate patients from their usual social support systems. Additionally, it is noted by Undrill et al. that some institutions primarily use risk assessments to manage liability, shifting the focus from patient-centered care to defensive medicine practices (termed “secondary risk management”), resulting in hospitalization of patients that may not have a clear benefit to treatment to mitigate assumption of risk [27]. One study used machine learning analysis of retrospective data from the Veterans Health Administration [28]. They found that in patients with a suicide attempt in the past day, psychiatric hospitalization was associated with a significant risk reduction in 12-month suicide attempt risk after discharge. However, in patients with suicide ideation or suicide attempt in the past 2–7 days, hospitalization did not reduce suicide attempt risk post-discharge. The authors conclude that there is a large amount of heterogeneity in terms of the impact hospitalization can have on the risk of subsequent suicide attempts and that an individual treatment rule could help to reduce suicide attempts [28]. Another paper conducted a retrospective analysis of adults treated by the National Health Service in England who died by suicide and compared rates in those who were cared for by a crisis resolution home treatment team to those who were psychiatric inpatients [29]. They found that the rate of suicides under the care of home treatment teams was higher than the average rate of suicide among psychiatric inpatients, and concluded that the home treatment team may not be appropriate for certain vulnerable people.

The infrequency of post-discharge suicide makes quantifying this effect challenging. However, Forte et al. attempted to quantify the level of suicide risk between those recently discharged from an inpatient hospitalization, compared to patients who had not recently been selected for psychiatric hospitalization [30]. They found that in the population who had recently (0–3 months) been discharged from inpatient hospitalization, they had an elevated level of risk compared to comparable controls who had not received hospitalization. While they indicate that much of this difference depends on mathematical effect, the data itself supports the theory that inpatient hospitalization may contribute in some part to the elevated risk of suicide.

### 3.4. Interventions and Strategies

Proposed and studied interventions to reduce the increased risk of suicide after discharge have focused on communication and contact with the patient post-hospitalization, though some interventions also involve education. Table 1 details the different interventions and their associated papers. A review article by Falcone et al. found that contact needs to be made with the patient post-hospitalization [31].

Brief contact interventions (BCIs) target feelings of social isolation and challenges in navigating community resources, which were identified as key modifiable risk factors. BCIs such as postcards, letters, phone calls, or crisis/green cards can be beneficial to patients in combination with emerging technologies and standard treatments [31]. A systematic review noted that researchers need to better describe and explain the mechanism by which BCIs function. It is still unclear as to which factors make follow-up contact modalities or methods more effective than others [32,39,40]. Increased social support and suicide prevention literacy appear to be the most common mechanisms of efficacious BCIs [39]. Generally, all effective interventions link patients to outpatient services, reduce feelings of social isolation, and help patients better navigate the available community resources [41].

One randomized control trial by Czyz et al. looked at the use of personalized communication post-discharge from the hospital to prevent adolescent suicidal behavior [36]. The patients were randomized to a Motivational Interview-Enhanced Safety Plan (MI-SP) alone or in combination with the use of text-based support during psychiatric hospitalizations, and then were re-randomized to a group that received telephone calls or no calls. The intervention was done across 1- and 3-month periods of discharge, when adolescents were at the highest risk for committing suicide. The MI-SP + Texts group experienced the same daily level of suicidal ideation with thoughts of method, intent, or plan. However, the MI-SP + Texts intervention lowered the intensity of suicidal urges and higher self-efficacy to engage in specific suicide coping assessed across follow-ups, reducing suicide attempts and suicidal behavior risk with an effect size of 0.2. The behavior change was not linked to or due to a change in suicidal ideation. They found that the booster telephone calls did not affect suicide attempts or behavior. It was noted that the interventions would likely be more effective if more tailored to each individual’s preferences, and that technology-enhanced interventions may help with effective continuity of care.

Another randomized control trial by Luxton et al. looked at BCIs in the context of US service members and veterans [32]. Veterans are around five times more likely to die by suicide within 12 weeks post-discharge from psychiatric hospitalization compared to the treatment population. Veterans’ risk is also 54 times higher than that of the general US population during the same period. Caring letters, a form of BCI, were trialed by psychiatrist Jerome Motto nearly five decades ago, successfully lowering the suicide rate significantly. The researchers updated the contact modality with emailed letters as opposed to physical letters and randomized two groups of patients. One group received 13 supportive emails over two years, while the other received standard care. The emails contained resources and were personalized with references to their hobbies, pets, or other details shared during the initial visit. There was no statistically significant difference between the groups on self-reported psychiatric readmissions, self-reported suicide attempts, or other measures for risk of suicide. It is unclear to the researchers why the study’s results differed from prior studies. The mechanisms of BCIs require further detailed research to inform future interventions.

Timing is crucial when reaching out to patients post-hospitalization. In the adult population in Korea, a study by Che et al. analyzed the timing of the first mental health outpatient care follow-up within 30 days after discharge for suicidal gestures [35]. A group of patients received outpatient care within 7 days of discharge, while another group received no care in the 30 days post-hospitalization. The hazard ratio for those who received care within 7 days was 0.82 compared to those who had not received care in 30 days after discharge. The sooner individuals with substance use disorder, schizophrenia, bipolar disorder, or depression receive outpatient follow-up care, the lower the risk of suicide. There is a strong recommendation for early follow-up care post-discharge from a psychiatric hospital.

Another potential intervention relates to the Safety Planning Intervention (SPI), which includes 6 steps that help the patient identify support persons and personal strategies in the event of a suicide crisis [38]. One study found that the SPI was associated with a reduction in suicide behavior across a 6-month follow-up period when it was implemented together with a telephone follow-up for patients who had presented to an ED for a suicide-related concern [33].

In countries with fewer psychiatric professionals, like China, education may play a more significant role. China has significantly less mental health care, with 5.47 psychiatric professionals per 10,000 people in China, compared to 38.78 in Korea, 11.9 in Japan, and 10–20 in European countries [37]. A qualitative analysis was conducted interviewing patients, their lay healthcare supporters (LHS), and mental health professionals in China. The patients believed that if their lay healthcare supporters were equipped with knowledge and skills for family-based interventions in psychiatric care and suicide prevention, they could significantly contribute to reducing post-discharge suicide risk. Considering the limited mental health resources in China, it could be beneficial for trusted family members or LHSs to be trained in delivering interventions.

Finland decreased the risk of suicide between 1995 and 2001 after a policy shift in the early 1990s to decentralize mental health care [24]. Patients were expected to use outpatient services provided by nongovernment or private organizations and treatment periods were shortened. The study compared patients in 1985–1991 and 1995–2001, and found there was a decrease in both one-week and one-year post-discharge suicide risk among individuals who were psychiatrically hospitalized. The risk of suicide within one year of hospital discharge significantly decreased among patients treated for schizophrenia when psychiatric hospital care was downsized. The deinstitutionalization process primarily benefits long-term patients transitioned into community care. It is possible that the local coordination of discharge practices was enhanced through decentralization.

There has been a reduction in suicidal ideation in hospitals and large health maintenance organizations that have comprehensive approaches to suicide prevention. In a study by Alexopoulos et al., 15 trained care managers offered algorithm-based recommendations to physicians and helped patients in primary care clinics adhere to their treatment over 24 months [34]. Compared to patients with usual care, the patients who received the intervention had a higher likelihood of receiving antidepressants and/or psychotherapy, with a 2.2 times greater decline in suicidal ideation.

Three evidence-based state-level policies were identified by the American Foundation for Suicide Prevention with support from The Pew Charitable Trusts for effective suicide prevention in healthcare settings [42]. The strategies involve training healthcare professionals in suicide prevention, ensuring mental health insurance coverage is equal to that of physical health care (mental health parity), and integration of suicide prevention efforts into primary care practices.

Some federal legislative advancements that were taken to allow for mental health to be incorporated into integrated and primary care include the Mental Health Parity Act of 1996, the Mental Health Parity and Addiction Act of 2008, and the Affordable Care Act of 2010 [43,44,45,46]. One study found there was an associated 5% reduction in suicide rates across the 29 states examined with mental health parity laws [47]. The Mental Health Parity Act (MHPAEA), the Affordable Care Act, and the Consolidated Appropriations Acts (CAAs) for fiscal years 2021 and 2023 laid the groundwork for mental health parity, strengthening and enforcing it—though suicide prevention was not explicitly addressed until the 2021 CAA [42]. As of now, 24 states and Washington, D.C., have laws requiring parity reporting. In states without such mandates, the 2021 Consolidated Appropriations Act permits state insurance commissioners to request data from insurers to verify compliance with parity laws. While this is the legislation, the US is far from parity, and the relationship between mental health treatment and reduced suicide has yet to be fully elucidated.

Researchers found three key elements that supported the implementation of all three suicide prevention state policies across the four states of Colorado, Montana, Oregon, and Vermont: committed leadership, strong collaboration between leaders and health care professionals, and ongoing financial support [42]. When these elements are combined, they can lead to effective and long-lasting suicide prevention efforts in health care settings. These findings align with insights from the literature review and expert interviews. However, three challenges that can create barriers to adopting and implementing suicide prevention policies are other pressing priorities, limited access to care and shortages of providers, and challenges in connecting prevention initiatives to measurable reductions in suicide rates.

## 4. Discussion

As our results revealed, there is robust data showing that the suicide risk for patients in the immediate time period after discharge from a psychiatric hospitalization is elevated. This is consistent across diagnoses, age groups, and different health systems. The timing of risk seems to matter more than the diagnosis alone. There is a lack of consensus about how to accurately identify, on an individual level, who is most at risk for suicide attempts or death by suicide after discharge. This could likely be driven by many different factors, including a lack of standardized and accurate risk assessment tools, indicating an area for future research [19]. While studies reliably indicate a high risk at the population level, precision is lost when narrowing the focus to assess individual-level risk, which further studies should explore and attempt to ameliorate [41]. Additionally, as individual prediction is weak, interventions that can be broadly applied, are low burden, and are timed to the immediate post-discharge period may be more fruitful than only targeting people who are labeled as being high-risk. Altogether, these findings suggest that post-discharge suicide risk may be better understood not as a narrowly predictable individual outcome, but as a shared period of vulnerability that warrants broad, time-sensitive prevention strategies.

Interventions that prioritized social support and suicide prevention education were most effective, though heterogeneous across studies [39]. BCIs in the form of text messages or phone calls timed within 6 months post-discharge were an automated way to alleviate feelings of social isolation, though the exact format that is most effective is not elucidated. Generally, the earlier the intervention within the 6-month span post-hospitalization, such as 1-week or 1-month post-discharge, the better the outcome for the patient. Similarly, early outpatient follow-up with a phone call had a positive effect in reducing risk when combined with a Safety Planning Intervention [33]. Importantly, these interventions do not require precise risk stratification to be somewhat effective, even if the effect sizes are modest [31,32,39,40,41]. This shift from individual-level prediction toward population-level prevention reflects the reality that suicide, while rare at the individual level, clusters within identifiable high-risk periods that can be addressed through universal or near-universal interventions. These findings suggest a public health approach in which we intervene in periods of vulnerability as opposed to predicting rare outcomes, just as seatbelts are mandated by law across the US to reduce mortality in car accidents that cannot be predicted beforehand. The relatively low cost, scalability, and feasibility of these interventions further strengthen their appeal for real-world implementation across diverse clinical settings.

Post-discharge suicide risk is exacerbated by weaknesses in the system. Issues such as unplanned or abrupt discharges, delays in follow-up care, or incomplete handoffs between inpatient and outpatient care can increase a patient’s risk. Different interventions focused on continuity, predictable follow-up, and access to community services can help address these system-level problems. System-level approaches may potentially reduce coercive or defensive practices around psychiatric hospitalization, especially those driven by liability concerns rather than patient-centered care [27,28]. In this way, system-level interventions may offer ethical as well as clinical benefits by supporting patient autonomy and continuity of care while reducing reliance on coercive or liability-driven decision making.

There are a few notable limitations of our data. For example, the literature often organizes patients through diagnoses, though psychiatric disorders are often concomitantly experienced; rarely does a patient present with one diagnosis. Many other disorders, like PTSD, OCD, and other diagnoses, are put under “other disorders” in many of these studies, which require further evaluation. Adolescents and adults are often analyzed within the same group, despite adolescents having different needs and experiencing different stressors that influence their suicidal behaviors compared to adults. Different healthcare systems have different access to psychiatric treatment. Some private hospitals may exclude patients at very high risk of suicide. Therefore, the findings may have artificially lower rates than socialized healthcare systems. The studies included in the interventions generally have smaller sample sizes, limiting the applicability of findings. It is important to keep in mind that small event counts can lead to low precision and high volatility in the data. Some of the studies also focused on specific populations, such as Veterans and US service members, which limits their applicability to other populations. Even though certain clinical variables are associated with suicide risk, the absolute risk for suicide is low. Thus, it is difficult to accurately predict suicide based solely on clinical variables [16]. Results from different studies varied significantly, and there may be a publication bias in which higher suicide rates were more likely to be published.

In the interest of space and focus, we limited our review in several important ways. Due to the implications of using a narrative review approach, our study selection, reproducibility, and interpretations of the synthesized conclusions are limited by the lack of inclusion or exclusion criteria and quantitative analysis. We have not attempted to distinguish post-hospitalization suicide risk in patients who were admitted voluntarily versus involuntarily. We also did not focus on the differences between sexes and race, but rather on the different interventions that have been actively implemented with all patients. We did not look at how other medical illnesses that were not psychiatric in origin have an impact on the patients’ suicidality. This would be a helpful subpopulation for researchers to study and inform future practice. We had not considered the effect of having firearms in the home as a variable, and it was not mentioned in the papers we reviewed. It may be helpful to see if patients with a firearm at home have a higher risk of suicide post-hospitalization and if any interventions could reduce that risk, such as education on safe storage in or out of the home, the use of Extreme Risk Protection Orders [48]. It may also be helpful to see how much patient disengagement could impact the efficacy of interventions. Finally, further research that highlights the experiences of suicide survivors themselves would broaden the field and provide much-needed insights into a difficult-to-understand phenomenon.

Therefore, it is crucial that we listen to the voices of those with lived experiences. Families and loved ones who have lost someone to suicide also have valuable insights into warning signs, care transitions, and missed opportunities that may not show up in the medical chart. Qualitative approaches that include interviews and psychiatric autopsy studies are important for understanding the post-discharge period and where systems may fail people. Future studies should attempt to address the heterogeneity in outcomes, definitions, and follow-up periods that made quantitative synthesis difficult in the current state of the field. Additionally, more work should be done that includes patient and survivor perspectives as well as implementation-focused trials. While no single intervention may “solve” post-discharge suicide, coordinated, system-oriented approaches focused on this narrow window of time may help us improve care and outcomes during one of the riskiest points in the trajectory of suicidal behavior.

## 5. Conclusions

Investigation into post-discharge suicide has demonstrated increased risk amongst psychiatric populations and settings, with those being diagnosed with any psychiatric illness at significantly increased risk. Much research is focused on elucidating specific factors that could better assess risk prior to discharge, as current risk assessments continue to be poor identifiers of those who will go on to engage in suicidal behavior [27]. Ultimately, broad, time-sensitive post-discharge prevention strategies may currently be more actionable and impactful to reduce risk than precise individualized risk prediction. In tandem, many have shifted focus towards the development of brief crisis interventions that specifically target this high-risk period. These interventions target social isolation and the inability to access care, which have specifically been demonstrated as modifiable risk factors for suicide. Interventions to increase engagement in mental health services, decrease time to follow-up, and structured institutional resources have shown promise to protect against deaths from suicide, though more studies are necessary [31,32,39,40]. Future reviews should include a more granular look at the effect of these interventions on populations diagnosed with comorbid psychiatric and substance use conditions, psychiatric disorders outside of the affective or psychotic domains (such as OCD/PTSD), those diagnosed with medical illnesses, voluntary vs. involuntary admission status, and adolescent vs. adult age. These future investigations will ultimately allow risk calculations to improve and allow for personalized interventions to prevent suicide during one of the highest-risk periods in an individual’s life.

## Figures and Tables

**Table 1:** Studies detailing various interventions trialed to reduce post-hospitalization suicide risk in multiple countries.

Study (Ref)	Design	Sample	Country	Population	Intervention	Methods	Key Outcomes
Luxton et al., 2014 [32]	Multi-site RCT	~1300	USA	Military and veteran patients at suicide risk	Caring letters post-discharge	Self-report, administrative records	Feasible, scalable suicide prevention approach
Stanley et al., 2018 [33]	RCT	1640	USA	Suicidal emergency department patients	SPI with follow-up calls vs usual care	Medical records, self-report	Reduced suicidal behavior; increased outpatient engagement
Alexopoulos et al., 2009 [34]	RCT	599	USA	Older adults with depression in primary care	Collaborative care model	Structured interviews, symptom scales	Sustained reduction in suicidal ideation and depression
Che et al., 2023 [35]	Retrospective cohort	>1 million	Republic of Korea	Psychiatric inpatients	Early outpatient follow-up after discharge	National claims data	Follow-up <=7 days associated with lower suicide risk
Czyz et al., 2021 [36]	Pilot SMART RCT	66	USA	Adolescents hospitalized for suicidality	Adaptive post-discharge intervention	Surveys, EMA, clinical outcomes	Feasible; reduced suicidal ideation; improved engagement
Fu et al., 2024 [37]	Qualitative study	44	China	Discharged psychiatric patients, caregivers, clinicians	Post-discharge suicide prevention perspectives	Interviews, thematic analysis	Identified care gaps; importance of family support
Stanley-Brown SPI [38]	Evidence-based intervention	NOT APPLICABLE	USA	Suicidal ED and inpatient patients	Safety Planning Intervention (SPI)	Clinical implementation data	Improved engagement; effective with follow-up
Milner et al., 2016 [39]	Systematic review	14 studies	International	Clinical populations with prior suicidality	Brief contact interventions	Systematic review	Benefits linked to connectedness and perceived care
Luxton et al., 2013 [40]	Review	NOT APPLICABLE	USA	Patients after suicidal crisis or psychiatric discharge	Brief follow-up contacts	Review of trials and observational studies	Associated with reduced suicide attempts and ideation
Falcone et al., 2017 [31]	Narrative review	NOT APPLICABLE	International	Recently discharged suicidal/high-risk psychiatric patients	Technology-based and outreach follow-up	Literature review	Improved continuity of care; potential reduction in suicidal behavior
Chaudhary et al., 2020 [41]	Narrative review	NOT APPLICABLE	International	Patients during transitions of care	Targeted transition-of-care interventions	Literature review	Structured follow-up may reduce post-discharge suicide risk

## Data Availability

No new data were created or analyzed in this study. Data sharing is not applicable to this article.
